# Study on the evolution of Chinese characters based on few-shot learning: From oracle bone inscriptions to regular script

**DOI:** 10.1371/journal.pone.0272974

**Published:** 2022-08-19

**Authors:** Mengru Wang, Yu Cai, Li Gao, Ruichen Feng, Qingju Jiao, Xiaolin Ma, Yu Jia

**Affiliations:** 1 Key Laboratory for Special Functional Materials of Ministry of Education, and School of Materials Science and Engineering, Henan University, Kaifeng, China; 2 Key Laboratory of Oracle Bone Inscriptions Information Processing, Ministry of Education of China, Anyang Normal University, Anyang, China; 3 School of Computer Science, Central China Normal University, Wuhan, China; 4 School of English Studies, Xi’an International Studies University, Xian, China; 5 Henan Museum, Zhengzhou, China; Newcastle University, UNITED KINGDOM

## Abstract

Oracle bone inscriptions (OBIs) are ancient Chinese scripts originated in the Shang Dynasty of China, and now less than half of the existing OBIs are well deciphered. To date, interpreting OBIs mainly relies on professional historians using the rules of OBIs evolution, and the remaining part of the oracle’s deciphering work is stuck in a bottleneck period. Here, we systematically analyze the evolution process of oracle characters by using the Siamese network in Few-shot learning (FSL). We first establish a dataset containing Chinese characters which have finished a relatively complete evolution, including images in five periods: oracle bone inscriptions, bronze inscriptions, seal inscriptions, official script, and regular script. Then, we compare the performance of three typical algorithms, VGG16, ResNet, and AlexNet respectively, as the backbone feature extraction network of the Siamese network. The results show that the highest F1 value of 83.3% and the highest recognition accuracy of 82.67% are obtained by the combination of VGG16 and Siamese network. Based on the analysis, the typical structural performance of each period is evaluated and we identified that the optimized Siamese network is feasible to study the evolution of the OBIs. Our findings provide a new approach for oracle’s deciphering further.

## 1. Introduction

Oracle bone inscriptions (OBIs), the ancient Chinese characters which are inscribed on cattle bones and turtle shells for making divinations, are the sources of Chinese characters and symbols of Chinese civilization for thousands of years. Since being unearthed in 1899, OBIs have gradually penetrated history, archeology [1], philosophy [2], paleontology [3], history of science and technology, and other related disciplines. On the other hand, OBIs have significant historical and cultural value, and their inheritance can provide much more cultural and academic insights into ancient civilization. Up till now, archaeologists have discovered about 4300 OBIs from various archaeological excavations, however, only about one-third of them have been interpreted completely. In fact, it is very difficult to decipher an oracle bone inscription due to its ages, unclear pronunciation and the disappearance of civilization caused by the loss of part of the evolution of Chinese characters, while the interpretation of OBIs requires a lot of preparatory work. Special researchers on OBIs, need to be familiar with all OBIs and master the basic information of all OBIs, especially the unexplained inscriptions. Therefore, the research of those unexplained OBIs has always been not only an important topic of OBIs-related research [4, 5], but also a historic vast project.

Since the beginning of Chinese civilization, the development of fonts of Chinese characters have undergone many stages of evolution, such as OBIs, Bronze Inscriptions, Seal Script, Official Script, and Regular Script. In the late Warring States period, before the appearance of Small Seal Script after first Emperor of Qin unified China, all Chinese fonts were known as ancient characters [6]. From the earliest times to the present day, the analysis and interpretation of OBIs are the same as that of other kinds of ancient characters, and the most basic method is the so called comparison method [7]. Researchers need to compare the unrecognized OBIs with known ancient inscriptions such as Bronze Inscriptions and Seal Scripts and infer the meaning of OBIs based on the latter information, to achieve the purpose of deciphering OBIs. Research on the evolution of Chinese characters is one of the significant methods to interpret OBIs. For example, Yang et al. [8] used Yolov2 to detect the roots of OBIs and analyze the evolution process of the oracle bone font structure. However, only a few studies have focused on the evolution of Chinese characters.

For this purpose, more recent studies have been reported to deep learning techniques to study OBIs. With the large-scale data on OBIs and the development of computer technology, especially artificial intelligence technology, it provides a new opportunity for interpreting unrecognized OBIs through artificial intelligence. For examples, Guo et al. [9] proposed a Hierarchical representation method, using Gabor transform and sparse expression, and then combined it with a Convolutional Neural Network (CNN) to identify OBIs; Lin et al. [10] utilized a single shot multibox detector to segment oracle bone rubbing images and recognize OBIs; Zhang et al. [11] designed an oracle bone text annotation software though deep learning based on a large-scale Oracle-8000 dataset; Sun et al. [12] used convolutional neural network for OBIs recognition and combined with Temporal-spatial Psychovisual Modulation (TPVM) to create a dual-view OBIs recognition system; Moreover, Zhang et al. [13] proposed a novel classification method to map character images to a Euclidean space where the distance between different samples can measure their similarities. The nearest neighbor rule can perform such classification; Gao et al. [14] introduced the VGG16 network which can be used to identify oracle variants, selected a set of uncertain recognition results, and then introduced a prior knowledge to integrate multi-domain methods to identify oracle variants, and so on.

In this contribution, we try to combine the overall structure of Chinese characters with FSL to provide a new approach for studying unrecognized OBIs [15]. Our starting point is that Chinese characters can be seen as a continuous and close-related system. And it is easy to find that an ancient character corresponds to a certain character in later generations, so it is possible and meaningful to find the correlation between them and use the existing rules to explain the various changes in the evolution of glyphs. The features of the current evolution of Chinese characters remain relatively regular in configuration. And the relationship between the glyph is continuous. For another reason, considering the lack of data and the type of Chinese character evolution, here we proposed the Siamese network based on a few samples to analyze the rule of Chinese character evolution.

## 2. Related works on FSL

Machine learning has had great success in data-intensive applications but is often inadequate in the face of small datasets. The recently emerged FSL method aims to solve this problem. FSL leverages prior knowledge and can quickly generalize to new tasks that contain only a few samples with supervised information. Inspired by human learning, Li et al. [16] proposed the concept of FSL, they argue that what has been learned about the old category can help predict the new category when there are only one or a few labeled samples of the new category. Various studies have shown that FSL is a close relationship in many fields, such as face recognition [17], handwritten font recognition [18], and image recognition [19]. FSL mainly includes those studies which focus on the following aspects: (1) Model-based fine-tuning, most refer to fine-tuning of parameters at the network layer. Howard et al. [20] proposed a universal language model called fine-tuning (ULMFit). For implementing transfer learning in fields like computer vision, ULMFit improves performance by 18–24% on multiple text classification tasks; (2) Based on data augmentation, the fundamental problem of FSL is that the sample size is too poor, which leads to the decrease of sample diversity. Data augmentation can be used to increase the diversity of features. Ren et al. [21] proposed the model-agnostic meta-learning (MAML) model for semi-supervised learning, which was improved three times based on the prototype network to improve the generalization ability of the algorithm; Schwartz et al. [22] proposed the Delta encoder, which can synthesize a few samples into a new one and trains the classifier through the new samples, but this method cannot significantly improve the classification boundary. According to this basis, Shen et al. [23] proposed that the fixed attention mechanism can be replaced by an uncertain attention mechanism, the two classifiers share parameters so that the high-level features guide the low-level features and optimize the network. (3) Based on transfer learning, Wang et al. [24] designed a regression network to obtain a transition between a small number of samples and a large number of samples, and map the multi-sample with excellent training effect to the few-sample with poor training effect; Jang et al. [25] proposed to use meta-learning to learn the weights of transfer feature maps and transfer layer weights. Solved the problem of transfer paths in transfer learning. (4) Based on similarity measures, in particular, Koch et al. [26] proposed a Siamese Neural network for single-sample image recognition, the main method is to minimize the loss of a pair of samples of the same category and maximize the loss of a pair of samples of different categories during the training phase; Vinyals et al. [27] proposed a Matching Network, which uses LSTM to map samples into a low-dimensional space. The key idea of the network is to map the image to an embedding space to achieve classification and detection effects; Shell et al. [28] proposed a Prototypical Network in which the distance between the same category is closer while the distance between different samples is farther; Sung et al. [29] proposed a Relation Network and divided the model into the embedded module with a 4-layer convolutional neural network and the relational module with comparability comparison. (5) Meta-learning, Santoro et al. [30] proposed a memory-augmented neural network (MANN), based on the Neural Turing Machine (NTM), and made a series of modifications to the training and storage read and write mechanisms; Sun Q et al. [31] proposed to initialize the parameters of the classifier randomly, and optimize it by the support set with the idea of MAML; All these studies show that small sample learning has certain advantages in multiple fields when the sample size is small.

In addition, in the process of feature extraction, the characteristics of the data need to be considered. We mainly focus on high-dimensional data such as images. For the mode of feature learning [32], it is considered that the convolutional neural network is currently the most used. The biggest feature of neural networks is the weight sharing between neurons and the sparse connections between convolutional layers [33], which can directly select large datasets when training the network. The AlexNet network [34] is an early proposed deep neural network, which mainly selects nonlinear activation functions. Subsequently, the VGG16 network [35] explores the relationship between the depth and performance of convolutional neural networks, which has strong extensibility; the Inception network [36] differs from the previous two networks in that it adds a structure called Inception, the main advantage of which is the reduction of training parameters; the ResNet network [37] introduces residual network structure, and it can realize that the accuracy does not decrease with the network deepening. These classic neural networks and other network structure models continue to emerge, resulting in the application of neural networks in various fields, such as natural language processing [38] and information decryption [39, 40], which bring great convenience to human life.

## 3. Data

**Fig 1:**
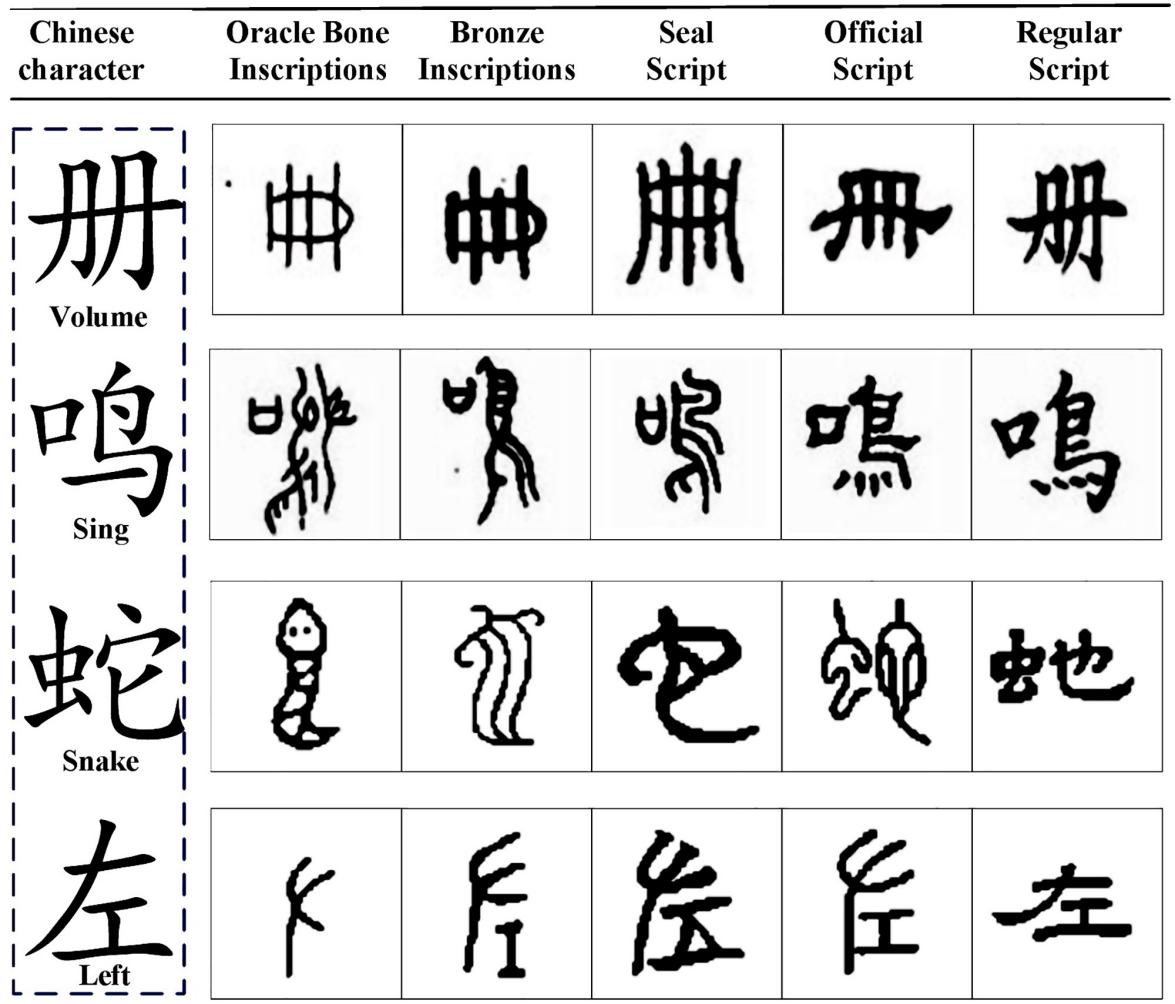
A sample of the data set and its contents.

In our study, we have established our own datasets. Our images are mainly from this Chinese master website (http://www.guoxuedashi.net/), All the raw images are freely available on this website. We first collected images of the evolution of existing Chinese characters on the website as the raw material and the main data source of the datasets. Secondly, we grayed the image and adjusted the size to 105×105 pixels. The dataset used in this work contains totally 972 categories. And each category contains OBIs, bronze inscriptions, seal script, official script, and regular script in the evolution of Chinese characters, therefore each category should include 5 images. We can easily find that the number of categories in each dataset is large but the number of samples in each category is small, as shown in Fig 1. Our datasets are available from Github (https://github.com/weirdo2310/OBIs-Evolution-of-Chinese-characters).

Next, we preprocess the images in the dataset. Image preprocessing operations mainly include grayscale processing and image size normalization. The grayscale processing adopts the weighted average method. The size normalization adopts the bilinear interpolation method, through which the size of the pictures in the Chinese character evolution dataset is normalized to 105×105 pixels. At present, the dataset is still increasing, and one of our purposes of this work is also to help other related research on OBIs identification.

## 4. Model architecture

We know that the evolution of Chinese characters is regular, and the characteristics of each period are nearly similar. And from the perspective of classification, we can explore the rules of the evolution of Chinese characters. Considering that the Siamese neural network is a network model of similarity measures, it can be used the learned metric to compare and match samples with new unknown categories. The model is used to deal with classification tasks with more categories and fewer samples in each category. Here we use the Siamese network to analyze the regularity of the evolution of OBIs. In this work, the model first uses the VGG16 network to extract features and then calculates the distance between the extracted features of two images. Eventually, the rules of Oracle’s evolution are explored. In the following subsections, we introduce the architecture of the VGG16 network, the Siamese network, the distance function, the loss function, and the training process, as well as the optimization of the algorithm successively.

### 4.1. The VGG16 network

For the Chinese character evolution dataset, we selected three classical convolutional neural networks to do experiments in this work. The experimental results show that the VGG16 network has excellent performance, and not only has high recognition accuracy but also has good local feature extraction and learning cycle. Therefore, VGG16 is selected as the main feature extraction network of the Siamese network.

**Fig 2:**
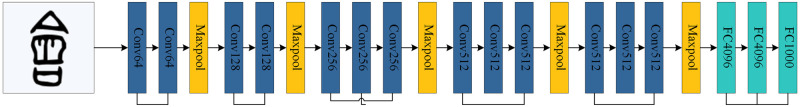
The VGG16 network can be used as the architecture of the feature extraction network.

The VGG16 network was involved in the ILSVRC-2014 competition and achieved the VGGNet achieved a 92.3% accuracy in the Top-5. Compared with previous models, the proposed model further broadens and deepens the network structure. Its core is five groups of convolution operations, and Max-Pooling space dimensionality reduction is performed between every two groups. Multiple consecutive 3×3 convolutions are used in the same group, and the number of convolution kernels increases from 64 in the shallower group to 512 in the deepest group. In the same group, the number of convolution kernels is the same. The convolution is followed by two full connection layers, followed by the classification layer. Here “Conv” represents the convolutional layer, “Maxpool” represents the max-pooling layer, and FC represents the full connection layer. Conv64 is 64 convolution kernels as shown in Fig 2.

We transform the major network of feature extraction to evaluate the performance of this method on our datasets. To effectively compare with our method, we use the same hyper-parameter and optimization algorithm. The AlexNet network structure has five layers of convolution layer and three layers of full connection layer, the width, and height of channels are reduced through convolution and pooling, and “padding = same” is welled set to ensure the same size of input and output. Compared with the AlexNet network, the obvious improvements of the ResNet network are the use a small size filter instead of a large one, two identical convolutions are set, the channel is raised and the width and height of the channel are reduced by pooling. The ResNet network is made up of residual blocks, which ensure that information can be transmitted over long distances.

The ResNet network has five models at different levels. Here we choose ResNet50, which has two basic blocks, namely Conv Block and Identity Block. The former is used to change the dimension of the network, while the latter is used to deepen the network. Despite the long jump connection, the computation is reduced, and the training can also be guaranteed to proceed smoothly. As seen clearly from the experimental results on our datasets in Table 1, we conducted the experiment based on the same environment in order to ensure the effectiveness of the comparative experiment. First, the evolution dataset of Chinese characters is randomly divided by the Bootstrap Resampling method [41], which can generate a new dataset without introducing new samples. Given a dataset *D* containing *m* samples, sample it and produce a dataset *D’*. Each time a sample is randomly selected from *D*, copied to *D’*, and then put it into the initial dataset *D*, so that the sample may still be sampled in the next sampling, and this process will be repeated *m* times, get a dataset *D’* containing *m* samples. Obviously, some samples in *D* will appear multiple times in *D’*, while others may not. In this work, about 36.8% of the samples in the original dataset will not appear in the new dataset, so there is about 36.8% of the samples in the original dataset *D* do not appear in the dataset *D’*, the *D* can be used as the training set and *D\D’* as the test sets. Here, since there are 4860 samples in our dataset, we take the value of *m* to be 4860. We conduct the random sampling with replacement for 4860 times and get training data with a size of 4860. It can be seen from Table 1 that among the precision, recall and F1 values, the algorithm combining the VGG16 network and the Siamese network performs more reasonably with the highest F1 value of 83.3%. The combined network is significantly ahead of the algorithms of AlexNet, ResNet-50 and the Siamese network. Among these, the experimental test results with different algorithms have proved that the combination of the Siamese network and the VGG16 network is more optimized in this study.

**Table 1:** Performance of three networks combined with Siamese networks.

Method	Precision	Recall	F1
**AlexNet + Siamese Network**	76.3%	79.6%	77.4%
**ResNet-50 +Siamese Network**	81.9%	82.7%	82.3%
**VGG 16 + Siamese Network**	84.1%	82.5%	**83.3%**

### 4.2. The Siamese neural network

For neural networks, one important feature is that they can extract features of images. We can naturally argue that if we need to compare the similarity of two images, we can utilize the backbone feature extraction network to perform feature extraction on the images that we input. The Siamese neural network is a neural network based on metric learning which contains multiple instances of the same model and shares the same architecture and weights. The parameters of the two networks are shared. The weights are shared so that it is impossible to map two similar images to a distant place in space. In addition, as the two networks are the same, the order of input pairs in the same pair has no effect on the embedding step. If the energy function is also symmetric concerning the two inputs, then the network is symmetric. Mapping the input to the target space by embedding function for similarity calculation using a metric function. Assuming that *x*_1_ and *x*_2_ are two input images, we use D(*x*_1_, *x*_2_) to indicate the similarity of two images. If D(*x*_1_, *x*_2_) is smaller, it means that the two images are also similar; while the two images are not of the same kind if D(*x*_1_, *x*_2_) is larger. So, a similar function is formulated as follows:

$$\left\{\begin{array}{l} D\left(x_{1},x_{2}\right)\leq \tau :\;\text{Similarity}\\ D\left(x_{1},x_{2}\right)>\tau :\;\text{Dissimilarity} \end{array}\right.$$ (1)

**Fig 3:**
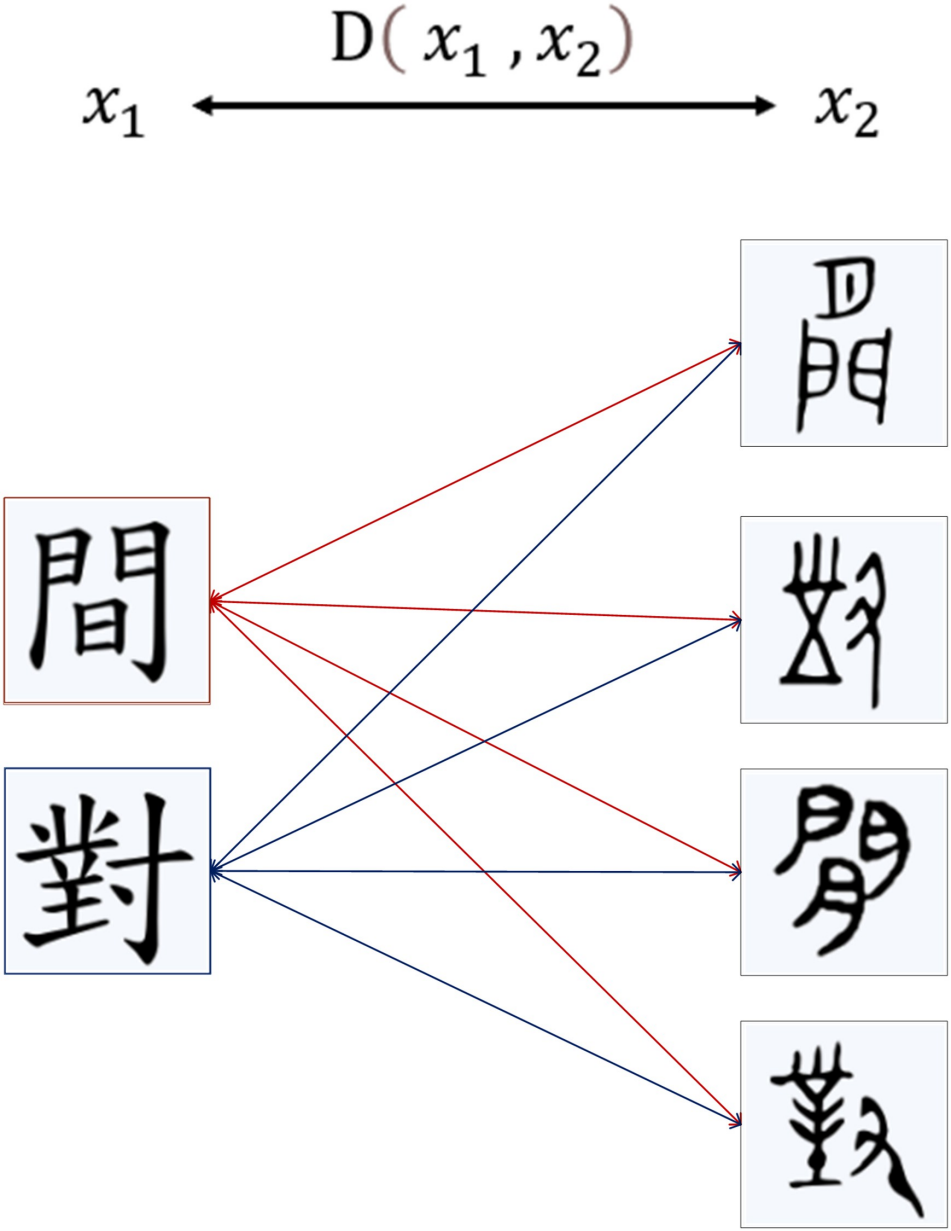
The two images are compared against each other.

As for the study of the evolution of Chinese characters in this work, only the similarity function between OBIs and Chinese characters in different periods needs to be calculated, and the target with the smallest D(*x*_1_, *x*_2_) is selected as the matching object. Certainly, if all D(*x*_1_, *x*_2_) are large, it means that it is not the evolution of the same Chinese character (Fig 3).

**Fig 4:**
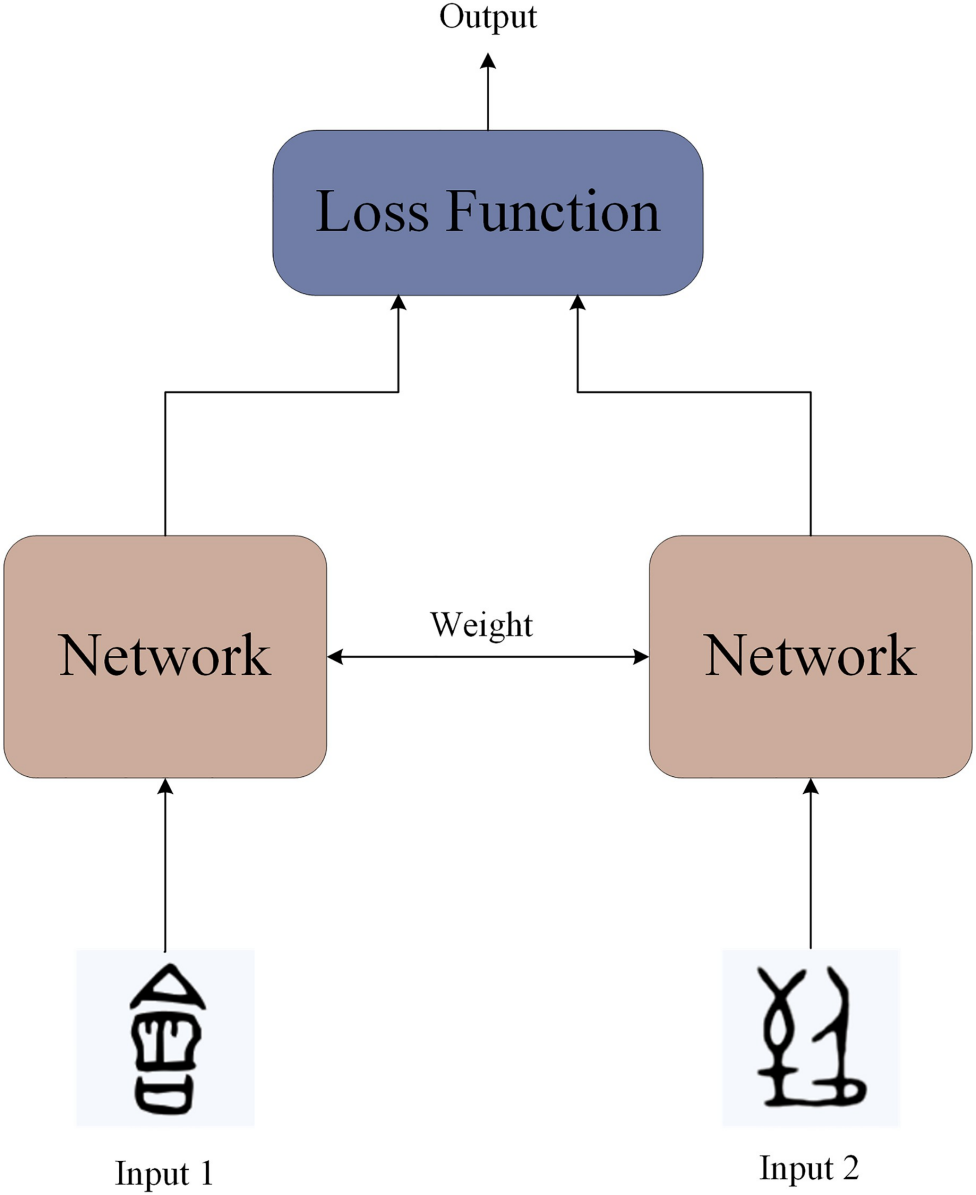
The architecture of the Siamese network.

The overall diagram of the Siamese network is shown in Fig 4. Establishing a simple convolutional neural network, including the convolution layer, pooling layer, and full connection layer. If an image passes through this network and finally gets the full connection layer, the full connection layer can be regarded as the encoding of the original image, which represents the key features of the original image. Each image is represented by each neuron in the full connection layer after passing through the Siamese network.

### 4.3. Overall framework

After using the VGG16 network as the extraction network, we get the multidimensional features and use the flattening function to flatten them into one dimension to get the one-dimensional vectors of the two inputs. The similarity function of two images *x*_1_ and *x*_2_ can be expressed by the norm of the difference between *f*(*x*_1_) and *f*(*x*_2_) of their respective FC layers:

$$\text{D}\left(\;x_{1}\;,\;x_{2}\right)=\left|f\left(x_{1}\right)-f\left(x_{2}\right)\right|$$ (2)

Then this distance is fully connected twice, the second time completely to a neuron. Sigmoid is taken for the result, and normalizes the output of each value to [0,1], which represents the similarity of the two input images. For two classification problems, the sigmoid activation function should be used with the binary cross-entropy function.

For our input samples, we define *X* = {*x*_1_, *x*_2_, …, *x*_*n*_}, The sigmoid unit first calculates the linear combination of *x*_1_, *x*_2_, …, *x*_*n*_, which is defined as *W*^*T*^*X*,

$$\text{δ}=\frac{1}{1+e^{-W^{T}X}}$$ (3)

We choose the cross-entropy loss function, and the multi-class cross-entropy loss function is defined as for Eq (3). Where *p*_*x*_ represents the probability that the sample is of class *x*. For the output of the Siamese network, we define that there are only positive and negative examples, and the sum of the probabilities of the two is equal to 1. The loss function definition for two classification tasks is simplified as Eq (4), where *h*_*ω*_ (*x*_*i*_) is the model prediction. This sample is a positive example of the probability, and *y*_*i*_ is the sample label. The cross-entropy is calculated here for each node, and each node has only two possible values. *y* = 1, when two inputs point to an image of the same type. *y* = 0, when two inputs point to different types of images. Then cross-entropy calculation is carried out between the network output result and the real label, which can be used as the final loss value. The loss function in this work is shown in the following formulas:

$$L\left(\omega \right)=E_{p}\left[-\text{log}\left(p_{x}\right)\right]$$ (4)

$$L\left(\omega \right)=-\frac{1}{n}\overset{n}{\underset{i=1}{\sum }}\left[y_{i}\;\text{log}h_{\omega }\left(x_{i}\right)+\left(1-y_{i}\right)\text{log}\left(1-h_{\omega }\left(x_{i}\right)\right]\right.$$ (5)

where *n* is the number of the samples, *p*_*n*_ is a true distribution, *q*_*n*_ is a non-true distribution. Data augmentation allows limited data to generate more equivalent data, enriches the distribution of training data, and increases the number and diversity of training samples. As well known, neural networks require a lot of parameters, and to make these parameters work correctly also requires a lot of data for training. But in this study, we need to explore the evolution rules Chinese characters between different period. Since the data of OBIs is limited at present, it cannot get reasonable results for using the network model. Therefore, we adopt one efficient method, namely data augmentation. Data augmentation is not only used to reduce the over-fitting phenomenon of the network but also used in the work which includes: horizontal flip, Rotation, Translation, and lens distortions (Table 2). The horizontal flip operation cannot flip all images in the horizontal direction, but flips randomly selected images every time. The rotation operation controls the rotation angle to 1º to 15º or -1º to -15º, since the labels of the data may no longer be retained with the larger rotation degree. When the image is panned, there will be some missing space in the image, which needs to be filled by setting "cval = 0" with a constant. Twisting operation is relatively easy. The image distortion can be completed by sinusoidal transformation of pixel coordinates and mapping of the transformed coordinates to corresponding coordinates. Accuracy parameter can reflect the possibility of real prediction made by the network model, and it should be the most concise and intuitive evaluation index. Also, it can more simply evaluate the performance of the method and eliminate the impact caused by the change of category distribution.

**Table 2:** Experimental results of data augmentation accuracy.

Data Augmentation	Accuracy
None	70.68%
Horizontal flip	72.51%
Flip, Rotation	75.34%
Flip, Rotation, Translation	79.53%
Flip, Rotation, Translation, Distort	82.67%

### 4.4. Experimental result and analysis

**Fig 5:**
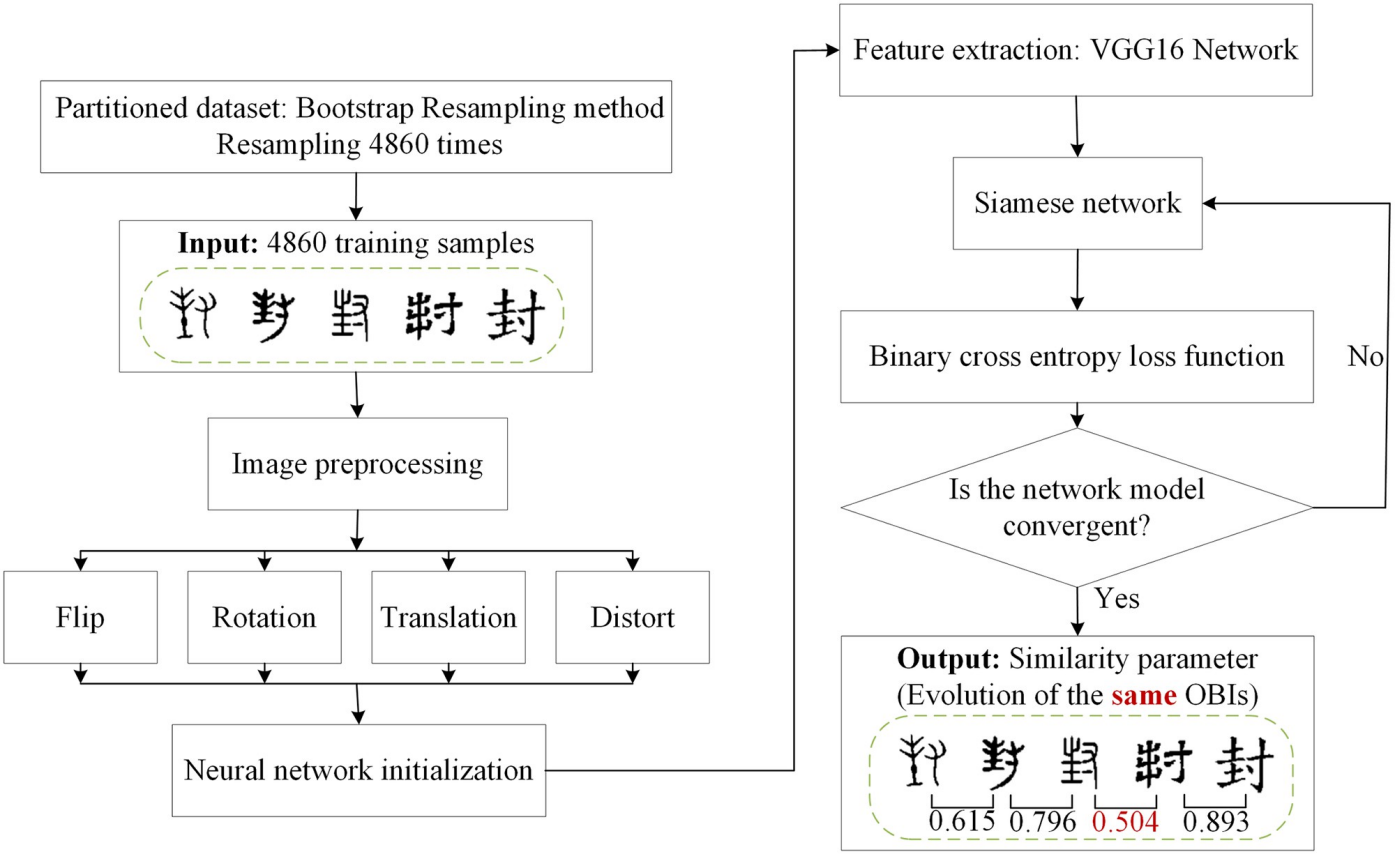
The Siamese network with the VGG16 network as the backbone feature extraction network predicts the evolution rules flow of Chinese characters.

In the Siamese network, our input includes two images, a pair of samples, the labels are only 0 and 1, in which label 1 is composed of the image of the same Chinese character, and label 0 is composed of two different images. The similar images indicate the evolution of the same character. We show the flow chart of the overall steps of the experiment in Fig 5. The process of training model includes four parts. Firstly, the Bootstrap Resampling method is used to divide the Chinese character evolution dataset. Secondly, the images in the training set are preprocessed, and then the neural network is initialized. The VGG16 network is used as the backbone feature extraction network of the Siamese network, and the extracted features are mapped to an n-dimensional hyperspace for similarity measurement calculation. Finally, the binary cross-entropy loss function is used. The result shows whether it is the evolution of the same Chinese character by the similarity between Chinese characters in adjacent periods.

**Fig 6:**
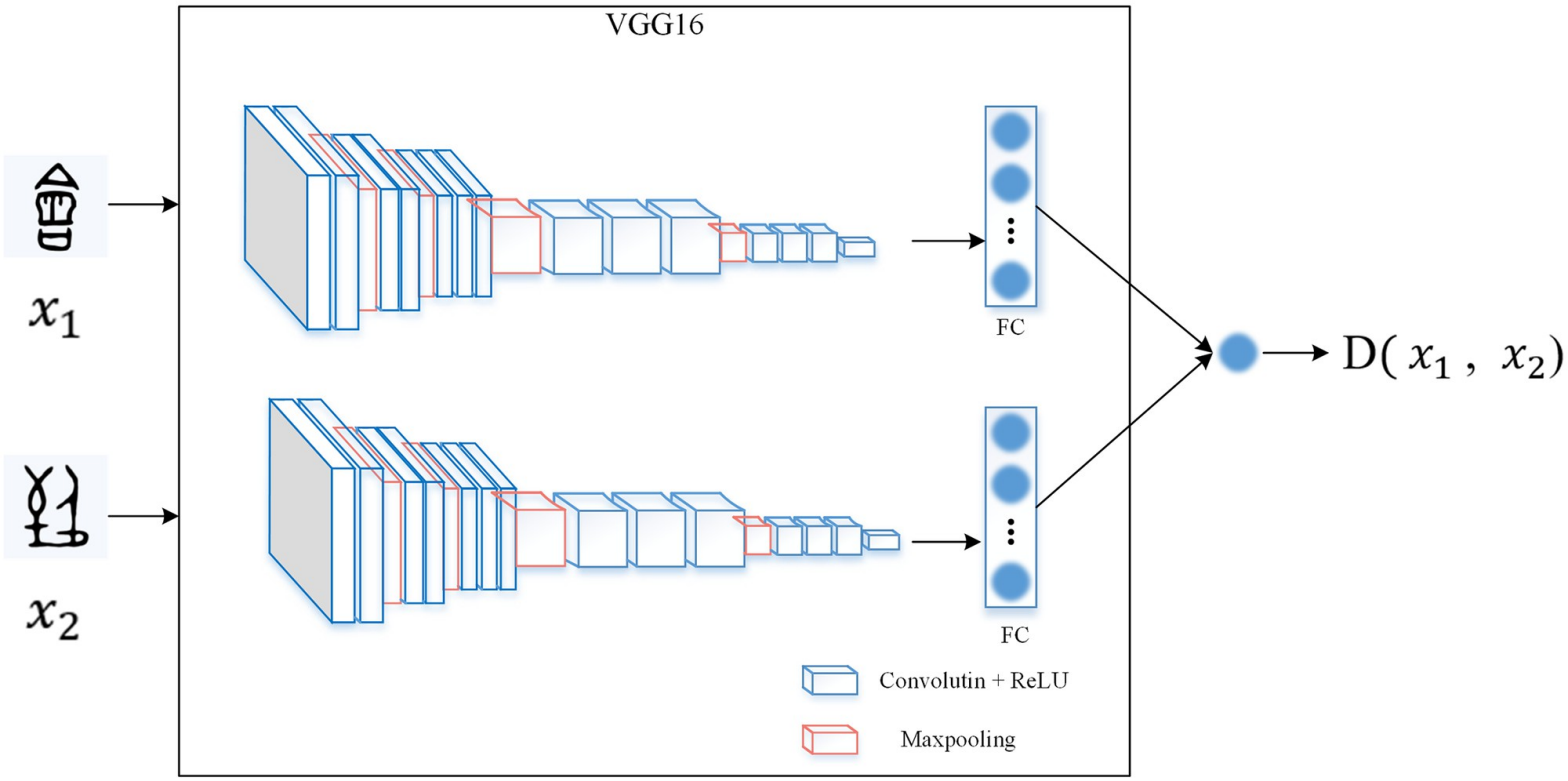
After feature extraction by the VGG16 network, a multi-dimensional feature is obtained and its similarity is measured.

**Fig 7:**
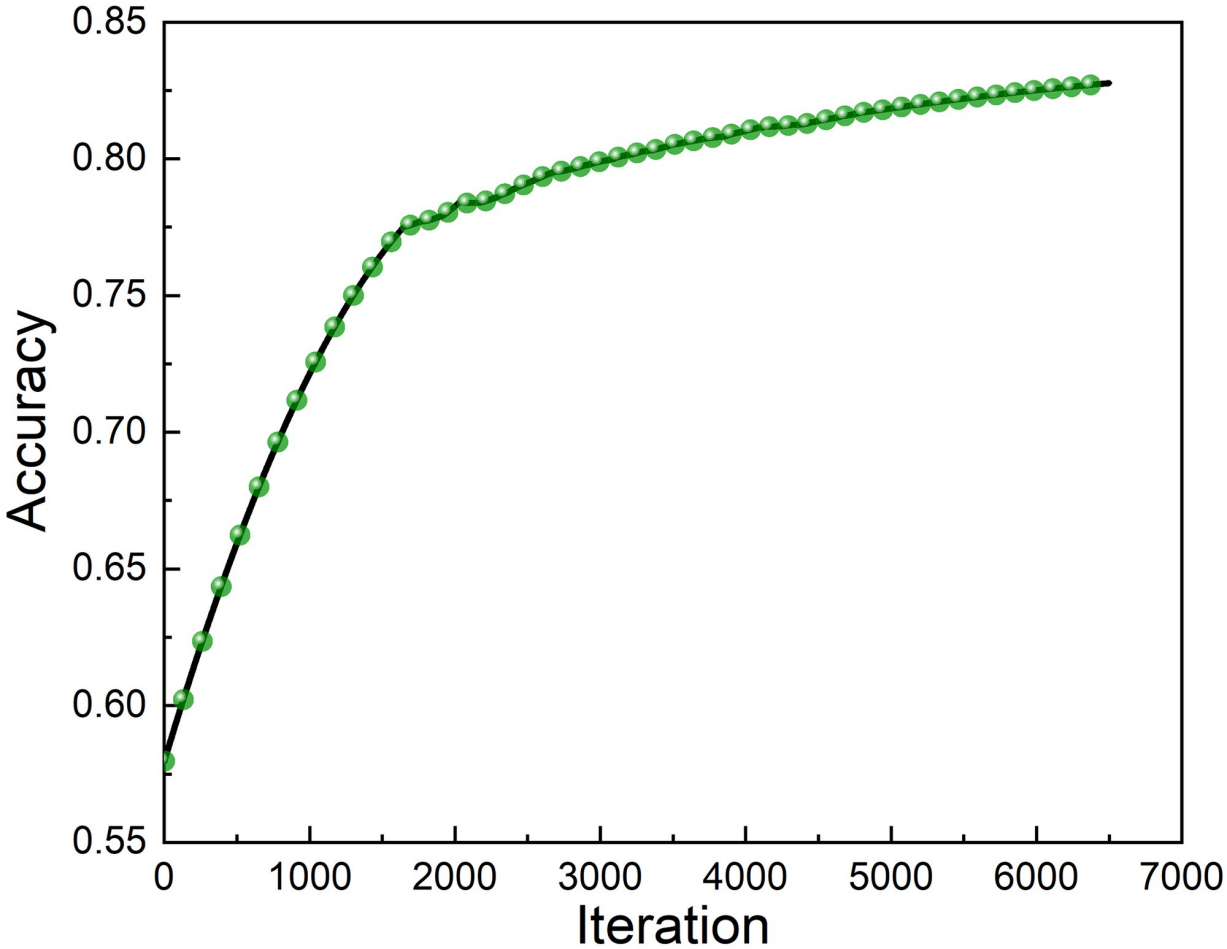
The accuracy rate of our training set.

And then we needed to select an image as the anchor, and record the anchor. A sample is randomly selected from the category where the anchor is located to form a positive sample, and then a sample is randomly selected from other classes that are not in the anchor class to form a negative sample (Fig 6). The training of the model uses 105×105×3 images. To prevent over-fitting during the training process, we can reduce the dimensions of each parameter. The method used is the early stopping method [42], it stopped the training when the performance of the model on the validation set declined. Only train on the training set, and calculate the error of the model on the validation set every cycle. Stop training when the model’s performance on the validation set declines. Finally, the parameters in the last iteration result are used as the final parameters of the model. Here, the accuracy rate is 82.67% in Fig 7. We use the trained network to make predictions.

**Fig 8:**
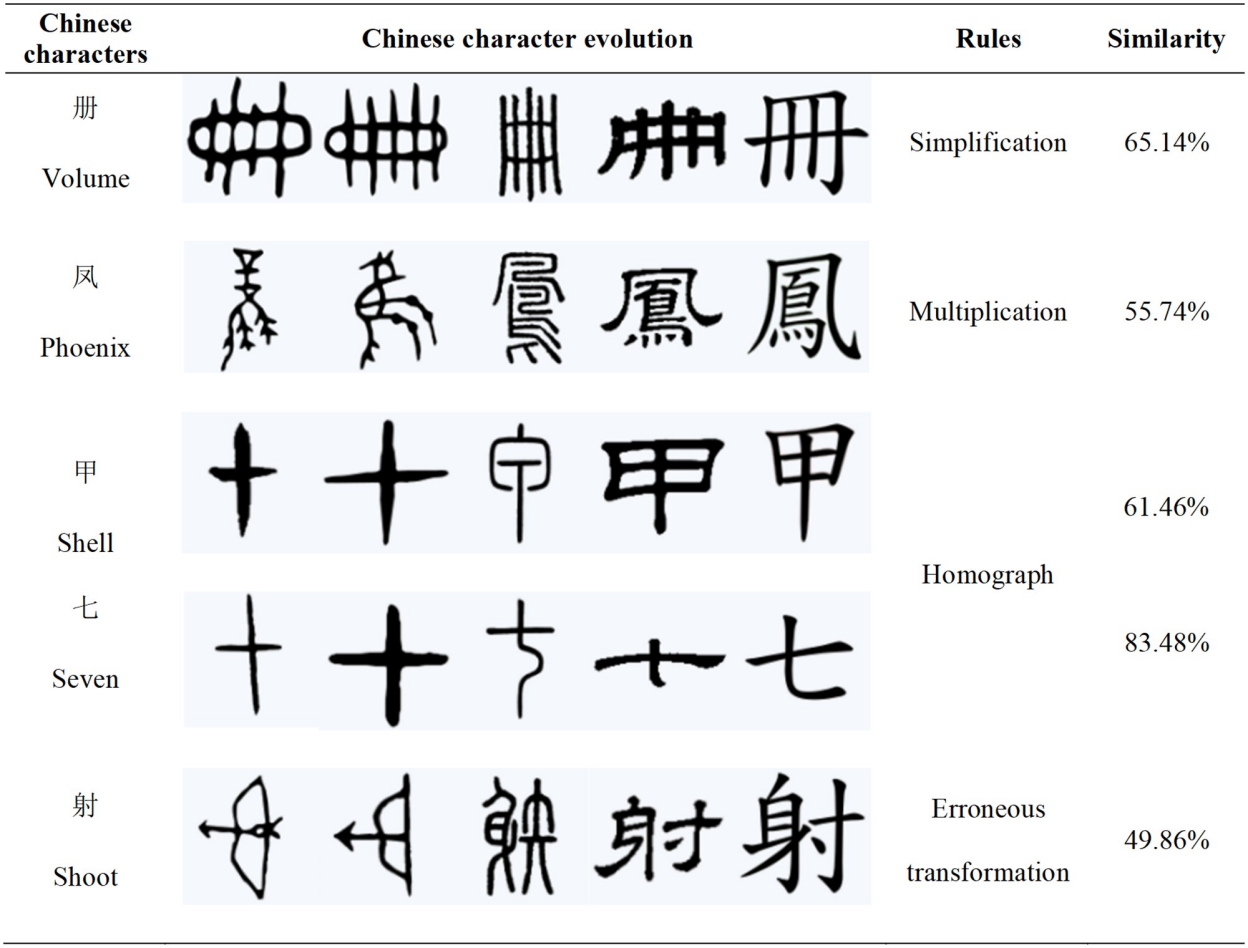
Detailed examples are given for four evolutionary rules.

Then we analyze them in proper order to facilitate writing and the original complex fonts were simplified. The official script opened up the modern writing era. In the process of simplification of Chinese characters, for example: "马", "册" and "贾" can be seen from the figures that their evolution is reflected in the simplification of the radicals, the simplification of the overall merger, the simplification of strokes, etc.; and the complication is to distinguish the meaning and pronunciation of Chinese characters, and to increase the number of times. "玉" is original "王", later people added a point to distinguish them; "鸡" has also changed from pictographic characters to "隹"; in the development and evolution of characters, affected by the specific language environment in which they are located, there will be many homographs appearing. The merging of characters specifically refers to the complete transfer of one or several characters to the corresponding character. We can see that "火" and "山", "甲" and "七" have the same shape in Fig 8, so the operation of merging characters is performed; another aspect that needs to be pointed out is that in the long-term development and use of Chinese characters. Inevitably, the wrong writing of Chinese characters will be inevitable. The propagation of errors is the corruption of Chinese characters. A typical example is "射" The specific data are shown in Table 3. In the process of the evolution of oracle bone inscriptions into Chinese characters, the Libian has crucial influence on the form of Chinese characters, which is called the watershed of Chinese characters. The forms of Chinese characters have changed a lot, which means that the similarity is not enough.

**Table 3:** The similarity of Chinese characters corresponds to the 4 evolution rules respectively.

Rules	The similarity of Chinese character evolution
**Simplification**	马: 76.44% 册: 65.14% 贾: 80.39% 对: 80.46% 首: 57.09%
**Multiplication**	玉: 82.54% 鸡: 76.74% 凤: 55.74% 肉: 58.90% 箕: 75.56%
**Homograph**	火: 91.30% 山: 94.69% 甲: 61.46% 七: 83.48% 匕: 79.04%
**Erroneous transformation**	射: 49.86% 丧: 75.52% 单: 79.94% 在: 49.41% 康: 53.41%

**Fig 9:**
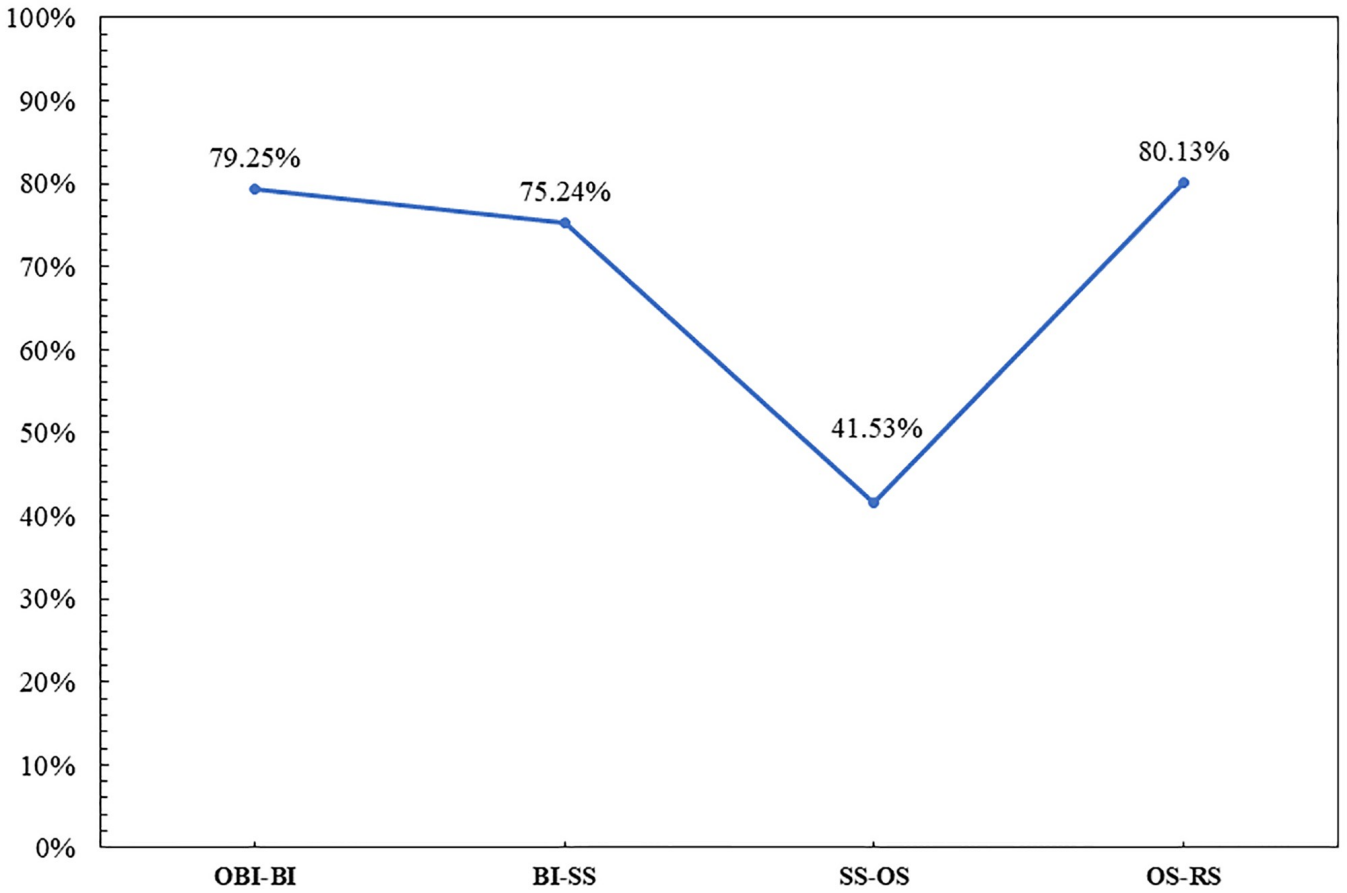
Accuracy between adjacent periods.

In general, we can divide the evolution of Chinese characters into four periods, which are from Oracle Bone Inscriptions to Bronze Inscriptions (OBIs-BIs), from Bronze Inscriptions to Seal Script (BIs-SS), from Seal Script to Official Script (SS-OS), and from Official Script to Regular Script (OS-RS), respectively. 200 groups of datasets were randomly taken out to analyze the similarity between adjacent periods, and the results are shown in Fig 9. We can learn that the accuracy is in line with the meaning of the evolution of Chinese characters in reality. The evolution from Official Script to Regular Script has the highest accuracy among all the periods, the main reason is that Regular Script preserves the structure of Official Script, and the character inheritance of this period is high, and there is no character fault. The second is from OBIs to Bronze Inscriptions the hieroglyphic degree between OBIs and Bronze Inscriptions is relatively high. The lowest accuracy is from seal script to official script, because this period experienced a script change from complexity to simplicity, and the writing speed from slow to fast. In all, in the whole evolution period of Chinese characters, the evolution similarity of adjacent periods is more accurate than that of the whole period. It should be noted that, although the VGG16 network performs the best in the whole process of Chinese character evolution, due to the low similarity of Chinese characters from the seal script to the official script period, the local feature extraction does not perform well in the feature extraction of the overall structure. This is also the limitation of the VGG16 network in the experiments.

## 5. Conclusion

In this work, we first established a Chinese character evolution dataset, and we used the Siamese network to analyze the evolution of OBIs with the dataset. Using the VGG16 network as the backbone feature extraction network, we have trained and optimized our dataset on the evolution of Chinese characters, and we obtained that the data optimization can promote the accuracy of the oracle’s deciphering. We show that there exists an inheritance relationship between the evolution of Chinese characters, and also the deformation of Chinese characters in each period is partially changed. Compared with the ResNet and AlexNet algorithm models, the VGG16 network has better performance in the local feature extraction of Chinese characters. The performance of the VGG16 network is not inferior to other networks in the case of a few samples [43]. In all, in finding the correlation between Chinese characters, our present method will be more efficient than traditional deciphering ways, which can provide a new approach for oracle’s deciphering.

## Supporting information

S1 File (RAR)

S2 File (PDF)

S1 Dataset (RAR)
