# Supplementary material for: Study on the evolution of Chinese characters based on few-shot learning: From oracle bone inscriptions to regular script
Source: PLoS One. 2022 Aug 19;17(8):e0272974. doi: 10.1371/journal.pone.0272974 (PMC9390942; doi:10.1371/journal.pone.0272974)
Supplement: S2 File — (PDF) [file pone.0272974.s002.pdf]

Authorship Statement

As corresponding author I, Yu Jia hereby confirm on behalf of all authors that:

The Chinese characters such as Oracle bone inscriptions studied in this paper are all public information and do not involve copyright issues. Not required permits obtained for accessing the artefacts.

Signature Yu Jia Date 2021/11/11
